# Associations between life satisfaction and hope with cognitive function and decline over 13 years: findings from the Whitehall II study

**DOI:** 10.1007/s10433-025-00892-8

**Published:** 2025-11-25

**Authors:** Amber John, Aysha Mohamed Rafik Patel, Roopal Desai, Emily Willroth, Natalie L. Marchant, Harriet Demnitz-King, Barbara Woodward-Carlton, Dorina Cadar, David Bartres-Faz, Rob Saunders, Georgia Bell, Aida Suarez Gonzalez, Darya Gaysina, Marcus Richards, Joshua Stott

**Affiliations:** 1 ADAPT Lab, Research Department of Clinical, Educational and Health Psychology, UCL, London, UK; 2 Psychological & Brain Sciences, Washington University in St Louis, St Louis, USA; 3 Division of Psychiatry, UCL, London, UK; 4 Brighton and Sussex Medical School (BSMS), University of Sussex, Brighton, UK; 5 Faculty of Medicine, Institute of Neurosciences, University of Barcelona, Barcelona, Spain; 6 CORE Data Lab, Centre for Outcomes and Research Effectiveness, Research Department of Clinical, Educational and Health Psychology, UCL, London, UK; 7 Dementia Research Centre, UCL, London, UK; 8 EDGE Lab, School of Psychology, University of Sussex, Brighton, UK; 9 MRC Unit for Lifelong Health and Ageing at UCL, London, UK; 10 Department of Psychology, University of Liverpool, Liverpool, UK; 11 London, UK

**Keywords:** Wellbeing, Life satisfaction, Hope, Cognitive function

## Abstract

Evidence indicates an association between wellbeing (e.g., purpose in life) and cognition over time. However, wellbeing is a multifaceted construct, and most research has focused on purpose in life and positive affect, with less research on other aspects of wellbeing. The aim of this study was to test associations between life satisfaction (LS) and hope with cognitive function and decline. Data were used from Whitehall II, a longitudinal cohort study of people employed by the British Civil Service. Measures of LS and hope were available at Wave 7, and cognitive function (phonemic/semantic verbal fluency, memory and inductive reasoning) at Waves 7, 9, 11, and 12. Linear mixed models were fitted to test associations between LS and hope with cognitive function and decline over 13 years. LS was positively associated with baseline cognitive function (overall cognition, verbal fluency, memory, and inductive reasoning) cross-sectionally but not with decline over time. Hope was positively associated with baseline overall cognition, phonemic fluency and inductive reasoning (but not semantic fluency or memory). Hope was associated with slower decline in inductive reasoning over 13 years. Findings contribute to better understanding of the temporal relationship between wellbeing and cognitive function from middle to older age. People with higher hope show lower baseline cognition and slower decline in inductive reasoning. People with lower LS show lower initial cognitive function and this difference is maintained over time. Although decline is not steeper for those with lower LS, they may reach the threshold for dementia earlier than those with higher LS.

**Supplementary Information:**

The online version contains supplementary material available at 10.1007/s10433-025-00892-8.

## Introduction

As a result of increasing life expectancies and population growth, improved understanding of issues affecting older people’s health is a global research priority (World Health Organization 2014). Maintaining cognitive health in ageing is one such priority. Cognitive decline is common during ageing, but the rate of decline experienced can vary significantly across different cognitive domains. Specifically, earlier and steeper declines may be observed in fluid cognitive domains such as episodic memory and processing speed, whereas crystallised domains such as knowledge and vocabulary are typically preserved with age (Salthouse 2010). Poorer cognitive function and faster cognitive decline are associated with reduced functional abilities and a range of mental and physical health problems, including dementia (Buckley et al. 2015; Alzheimer’s Society 2018). It is important to understand factors associated with cognitive function, as this may offer insights into potential interventions that may slow cognitive decline prior to the onset of dementia and improve mental and physical health in later life. If the onset of dementia were delayed by just one year, this could potentially translate to a reduction of up to 9.2 million cases worldwide by 2050 (Brookmeyer et al. 2007).

Wellbeing has been defined as a combination of psychological health and effective functioning (Huppert 2009). This can include finding life enjoyable and pleasurable, as well as the pursuit of self-growth and fulfilment. Recent systematic reviews and meta-analyses have provided early evidence to suggest that wellbeing may be associated with better cognitive outcomes over time, though this research area remains in its infancy (Bell et al. 2022a; Willroth et al. 2023). There are multiple pathways which may underlie associations between wellbeing and cognitive function and decline over time. First, higher levels of wellbeing are associated with a range of healthy lifestyle behaviours, such as social and physical activities (Willroth et al. 2024), which are in turn associated with better cognitive outcomes (Livingston et al. 2024). Second, wellbeing may contribute to greater cognitive resilience to dementia-related neuropathology (Willroth et al. 2022). Third, both may be linked by a physiological common cause. As such, there are several direct and indirect pathways through which wellbeing may be associated with cognitive outcomes over time.

However, wellbeing is multifaceted and is made up of multiple inter-related but distinct constructs (Willroth 2023). Evidence suggests that different aspects of wellbeing may have differential associations with cognitive function, with stronger effects generally observed for eudemonic (defined as living in a way that is line with an individual’s potential, e.g., purpose in life) compared with hedonic (defined as experiencing life as pleasurable, happy, and satisfying, e.g., positive affect) wellbeing (Bell et al. 2022b, a). To date, the majority of research has focussed on purpose in life (Boyle et al. 2010; Windsor et al. 2015; Kim et al. 2019; Nakanishi et al. 2019; Dewitte et al. 2021; Lewis and Hill 2021; Shin et al. 2022) and positive affect (Bishop et al. 2011; Danhauer et al. 2013; Berk et al. 2017; Castro-Schilo et al. 2019; Nakanishi et al. 2019; Hittner et al. 2020), and many other aspects of wellbeing remain comparatively under-explored. More work is needed to better understand associations between other domains of wellbeing and cognitive function and decline over time. This study will focus on two specific aspects of wellbeing: hope and life satisfaction.

Hope is commonly defined as the desire for a goal that is possible but uncertain, and a comprehensive conceptual framework of hope has also been recently developed (Pleeging et al. 2022). Hope is an important construct within wellbeing and evidence has shown that hope in older adulthood is associated with a range of positive long-term outcomes, including better physical health, fewer chronic conditions, lower rates of cancer, fewer sleep problems and lower all-cause mortality (Long et al. 2020). However, limited evidence exists on the association between hope and cognitive function or decline. One study reported a positive cross-sectional association between hope and cognitive function (Waldman-Levi et al. 2020), however the longitudinal association between the two is not yet known. This has been identified as an under-researched but promising area for future study (Bell et al. 2022a).

Life satisfaction can be defined as how a person feels about their life as a whole, and can include level of satisfaction with different domains of life (e.g., work, relationships, health, etc.). Similarly to hope, life satisfaction in older adulthood has also been associated with positive outcomes, including better physical health (including lower risk of pain, fewer chronic conditions, higher self-rated health), fewer sleep problems, more physical activity, and lower all-cause mortality (Collins et al. 2009; Kim et al. 2021). Compared with hope, there have been more studies focusing on associations between life satisfaction and cognitive function, though the majority of this research has been cross-sectional in nature. This research has been mixed, with some studies reporting cross-sectional associations (Jones et al. 2003; Requena et al. 2009; Zahodne et al. 2018) and others reporting no relationship (Bishop et al. 2012; Wettstein et al. 2015). Less is known about associations between life satisfaction and cognitive function over time. One study reported that higher scores on items based on life satisfaction and satisfaction with ageing were associated with slower decline in perceptual speed over 13 years (Gerstorf et al. 2007). Another study showed that reductions in life satisfaction were associated with future decreases in multiple cognitive domains, including spatial cognition, processing speed, and verbal working memory (Zainal and Newman 2022). However, a further two studies showed no associations between life satisfaction and later cognitive state (Nakanishi et al. 2019) or in change in executive function over time (Ihle et al. 2021). Related to cognitive function, other studies have reported longitudinal associations between life satisfaction and risk of MCI or dementia (Rawtaer et al. 2017; Zhu et al. 2022).

In light of these contrasting findings, the primary aim of this study is to test associations between life satisfaction and hope with cognitive function and decline in a range of domains (verbal fluency, memory, inductive reasoning, and overall cognitive function) over a 13-year period.

## Methods

### Participants

Participants were from the Whitehall II Study (Marmot and Brunner 2005), a longitudinal cohort, which originally comprised 10,308 men and women aged 35–55 who were employed by the British Civil Service. Data have been collected approximately every 2–5 years since the baseline in 1985–1988. Assessments have alternated between a postal questionnaire only and a postal questionnaire and clinical examination. Written informed consent was obtained from cohort members before participation and ethical approval was granted by the University College London Ethics Committee. To date there have been 12 waves of data collection. Detailed information about the Whitehall II Study has been published elsewhere (Marmot and Brunner 2005). Participants were included in analyses if: (1) data on life satisfaction and hope were available at analytic baseline (Wave 7); (2) at least one valid cognitive measurement was available at follow up. Participants were excluded from adjusted models if they had missing data on key covariates.

### Measures

#### Wellbeing

Two measures of wellbeing (hope and life satisfaction) were selected from Wave 7 (2003–2004), which was used as the analytic baseline for the present research. Hope was measured using a 6-item scale adapted from the Beck Hopelessness Scale (Cronbach’s alpha = 0.80) (Beck et al. 1974; Singh-Manoux et al. 2003). This included both positively worded items, such as “I look forward to the future” and negatively worded items, such as “Things look bleak”. Participants responded to each item using a 5-point Likert scale (ranging from absolutely disagree to absolutely agree). Negatively worded items were reverse scored so higher scores represent greater levels of hope. A total score was derived by calculating a sum score for the 6 items (possible range: 0–24). Life satisfaction was measured using a single item asking participants about their satisfaction with life as a whole and was scored using a 7-point Likert scale (ranging from very dissatisfied to very satisfied). Higher scores represent greater satisfaction.

#### Cognitive function

Cognitive functioning was measured with phonemic and semantic verbal fluency, memory, and inductive reasoning at Waves 7, 9, 11, and 12 (13 years follow up. Age range 50–74 at analytic baseline). Cohort members were asked to write down as many words beginning with the letter “S” (**phonemic verbal fluency**) and as many animal names (**semantic verbal fluency**) as possible within a one-minute period (Borkowski et al. 1967). **Memory** was assessed using a word recall task, in which a series of 20 words were presented and cohort members were asked to write as many words as they could remember within a two-minute period. **Inductive reasoning** was assessed using the Alice Heim 4-I (Heim 1970), in which cohort members are asked to complete a series of 65 verbal and mathematical reasoning questions which increase in level of difficulty within an allotted period of 10 min. Based on a procedure commonly used in previous research using this data, **overall cognitive scores** were derived by calculating z scores for each of the individual cognitive tests described above, and then calculating a mean of these scores (Singh-Manoux et al. 2012; Rusmaully et al. 2017; Krause et al. 2019).

#### Covariates

Covariates were age, sex, ethnicity (coded as White or Minority ethnic), marital status (coded as Married, Cohabiting, Single, Divorced, Widowed), number of years in education, and depression (whether a cohort member reported depression or nervous problems in the past 2 weeks, coded as Yes or No). All covariates were selected from the study baseline (Wave 7), except for education, which was available at an earlier time point (Wave 5).

### Statistical analyses

#### Main analyses

Demographic information was reported for the analytic sample included in fully adjusted models. Missing data were explicitly examined and reported on, and the sample included in fully adjusted models were compared to the sample excluded due to missing data based on key variables and covariates, using chi square or t-tests as appropriate. This was to reflect cumulative patterns of attrition across the full follow up period.

Linear mixed models were fitted to cognitive data to model trajectories from Wave 7 through to Wave 12 over a period of 13 years. The intercept and slope were fitted as random effects to allow for individual variation in cognitive function and decline. An unstructured covariance structure was assumed. Hope and life satisfaction at Wave 7 were included in models as fixed effects to predict cognitive function at analytic baseline. Interaction terms between hope and life satisfaction with time (in years) were included to predict cognitive slope. Two models were run: 1. Unadjusted (Model 1); 2. Adjusted for key covariates (age at baseline, sex, ethnicity, marital status, education, and depression) (Model 2). Models were run separately for hope (Model 1a and 2a) and life satisfaction (Model 1b and 2b), to avoid issues of multicollinearity. Missing data were dealt with using full information maximum likelihood (FIML) in all models. All analyses were conducted using Stata v17.

#### Supplementary analyses

To ensure that presence of dementia did not have any disproportionate effect on the results, supplementary analyses were conducted excluding people diagnosed with dementia at any point during the study (n = 251). In addition, supplementary analyses were also run excluding people who died during the follow up period (n = 1,799). Additional supplementary analyses were run including age bands as categorical age bands and their interaction with time, to account for non- linear effects of age of cognitive function and decline.

Analyses were also run including sex interaction terms to test for sex differences in associations. Where there were significant interactions, models were run stratified by sex. Finally, analyses were run including interaction terms to test for age differences in associations. Models were run stratified by age group (age < 65, age 65 +) where significant interaction terms were observed.

## Results

### Demographics and missing data

The analytic sample (n = 5,716) is described in Table 1. This sample includes participants with data available on at least one measure of cognitive function across time and data for hope, life satisfaction and covariates, making them eligible for inclusion in at least one of the adjusted models (hope n = 5,766; life satisfaction n = 5,772).

**Table 1:** Demographics of analytic sample, N = 5,716

	N (%) or mean (SD)
Age, N (%)	
50–54	1,063 (18.60)
55–59	1,728 (30.23)
60–64	1,220 (21.34)
65–69	1,164 (10.36)
70–74	541 (9.46)
Sex, N (%)	
Male	4,129 (72.24)
Female	1,587 (27.76)
Ethnicity, N (%)	
White	5,315 (92.98)
Ethnic minority	401 (7.02)
Marital status, N (%)	
Married/Cohabiting	4,363 (76.33)
Single	709 (12.40)
Divorced	406 (7.10)
Widowed	238 (4.16)
Education, Mean (SD)	15.03 (4.17)
Depression, N (%)	
No	4,573 (80.00)
Yes	1,143 (20.00)
Hope, Mean (SD)	18.57 (4.57)
Life satisfaction, Mean (SD)	5.5 (1.57)

Briefly, the sample comprised higher proportions of men (72.24%) and people from white ethnic backgrounds (92.98%) than would be expected from general population estimates. Additionally, the majority of the sample reported being married or cohabiting at baseline (76.33%) with an average of 15.03 years in education. Approximately 20% of the sample reported experiencing depression/nervous problems in the last 2 weeks.

The analytic sample (n = 5,716) was compared to the sample excluded due to missing data (n = 4,595) on key variables and covariates (Supplementary Table 1). The analytic sample was relatively smaller than the number of participants who initially took part at Wave 1, because many participants had already dropped out of the study prior to the baseline used for this study (Wave 7). Compared to the analytic sample, the latter were more likely to be older, women, from an ethnic minority background, single, divorced or widowed, have lower educational attainment, and to report experiencing depression. Those with missing data also reported lower life satisfaction and hope. They also obtained lower cognitive scores (for all cognitive domains).

### Associations between hope and life satisfaction with cognitive function and decline

The unadjusted models for hope (Table 2) showed that higher levels of hope were associated with higher baseline cognitive scores in all domains. Overall cognition: n = 6,399, b = 0.01, SE = 0.002, *P* < 0.001. Phonemic fluency: n = 6,389, b = 0.06, SE = 0.01, *P* < 0.001. Semantic fluency: n = 6,390, b = 0.04, SE = 0.01, *P* < 0.001. Memory: n = 6,387, b = 0.01, SE = 0.01, *P* = 0.02. Inductive reasoning: n = 6,396, b = 0.23, SE = 0.03, *P* < 0.001). Hope was also associated with slower decline in inductive reasoning over time (b = 0.003, SE = 0.001, *P* = 0.05), but not with rate of decline for other cognitive functions (Overall cognition: b = 0.00004, SE = 0.0001, *P* = 0.75. Phonemic fluency: b = − 0.0004, SE = 0.001, *P* = 0.62. Semantic fluency: b = 0.0002, SE = 0.001, *P* = 0.74. Memory: b = − 0.001, SE = 0.001, *P* = 0.24).

**Table 2:** Unadjusted linear mixed models testing associations between hope with cognitive function and decline

Overall cognition (N = 6,399)	
Time	− **0.01 (0.002), < .001**^**a**^
Hope	**0.01 (0.002), < .001**
Hope X Time	0.00004 (0.0001), .75
Random intercept variance	0.51 (0.01)
Random slope variance	0.001 (0.00003)
Intercept-slope covariance	− 0.0002 (0.0004)
Verbal fluency: Phonemic (N = 6,389)	
Time	− **0.09 (0.02), < .001**
Hope	**0.06 (0.01), < .001**
Hope X Time	− 0.0004 (0.001), 0.62
Random intercept variance	11.45 (0.30)
Random slope variance	0.02 (0.002)
Intercept-slope covariance	− 0.02 (0.02)
Verbal fluency: Semantic (N = 6,390)	
Time	− **0.09 (0.01), < .001**
Hope	**0.04 (0.01), < .001**
Hope X Time	0.0002 (0.001), 0.74
Random intercept variance	10.32 (0.26)
Random slope variance	0.007 (0.001)
Intercept-slope covariance	− 0.02 (0.01)
Memory (N = 6,387)	
Time	− **0.10 (0.01), < .001**
Hope	**0.01 (0.01), 0.02**
Hope X Time	− 0.001 (0.001), 0.24
Random intercept variance	2.98 (0.09)
Random slope variance	0.004 (0.001)
Intercept-slope covariance	− 0.03 (0.007)
Inductive reasoning (N = 6,396)	
Time	− **0.23 (0.03), < .001**
Hope	**0.23 (0.03), < .001**
Hope X Time	**0.003 (0.001), 0.05**
Random intercept variance	112.02 (2.18)
Random slope variance	0.08 (0.005)
Intercept-slope covariance	0.12 (0.08)

^a^ Presented as: b (SE), *p* <.05

^*^Note: The overall cognition measure is expressed in standard deviation units (due to calculation of z scores), whereas the individual cognitive outcomes are reported using original units

The fully adjusted models for hope (Table 3) showed that this was associated with overall cognitive function (n = 5,766, b = 0.01, SE = 0.002, *P* = 0.004), phonemic fluency (n = 5,760, b = 0.04, SE = 0.01, *P* = 0.001), and inductive reasoning (n = 5,765, b = 0.08, SE = 0.03, *P* = 0.004), but not semantic fluency (n = 5,761, b = 0.01, SE = 0.01, *P* = 0.34) or memory (n = 5,758, b = 0.01, SE = 0.01, *P* = 0.19) at analytic baseline. Levels of hope were associated with slower rates of decline in inductive reasoning (b = 0.003, SE = 0.001, *P* = 0.03), but not with rate of decline in other cognitive domains (Overall cognition: b = 0.0001, SE = 0.0001, *P* = 0.63. Phonemic fluency: b = − 0.0002, SE = 0.001, *P* = 0.84. Semantic fluency: b = 0.001, SE = 0.001, *P* = 0.51. Memory: b = − 0.0004, SE = 0.001, *P* = 0.48).

**Table 3:** Adjusted linear mixed models testing associations between hope with cognitive function and decline

Overall Cognition (n = 5,766)	
Time	− **0.01 (0.002), < .001**^**a**^
Hope	**0.01 (0.002), 0.004**
Hope X Time	0.0001 (0.0001), 0.63
Ethnicity	− **0.90 (0.03), < .001**
Sex	− 0.03 (0.02), 0.09
Age	− **0.22 (0.01), < .001**
Marital status	
Married/Cohabiting	*REF*
Single	− **0.13 (0.03), < .001**
Divorced	− **0.09 (0.03), 0.004**
Widowed	− **0.10 (0.04), 0.01**
Education	**0.03 (0.002), < .001**
Depression	− 0.01 (0.02), 0.69
Random intercept variance	0.36 (0.008)
Random slope variance	0.0005 (0.00004)
Intercept-slope covariance	− 0.003 (0.0004)
Verbal fluency: Phonemic (N = 5,760)	
Time	− **0.09 (0.02), < .001**
Hope	**0.04 (0.01), 0.001**
Hope X Time	− 0.0002 (0.001), 0.84
Ethnicity	− **2.76 (0.18), < .001**
Sex	**0.25 (0.11), 0.02**
Age	− **0.82 (0.04), < .001**
Marital status	
Married/Cohabiting	*REF*
Single	− **0.75 (0.14), < .001**
Divorced	− **0.44 (0.18), 0.01**
Widowed	− **0.61 (0.24), 0.01**
Education	**0.10 (0.01), < .001**
Depression	0.05 (0.13), 0.70
Random intercept variance	9.76 (0.28)
Random slope variance	0.02 (0.002)
Intercept-slope covariance	− 0.06 (0.02)
Verbal fluency: Semantic (N = 5,761)	
Time	− **0.09 (0.01), < .001**
Hope	0.01 (0.01), 0.34
Hope X Time	0.001 (0.001), 0.51
Ethnicity	− **3.89 (0.16), < .001**
Sex	0.10 (0.09), 0.29
Age	− **0.86 (0.03), < .001**
Marital status	
Married/Cohabiting	*REF*
Single	− **0.54 (0.13), < .001**
Divorced	− **0.46 (0.16), 0.004**
Widowed	− **0.54 (0.21), 0.01**
Education	**0.11 (0.01), < .001**
Depression	− 0.03 (0.11), 0.81
Random intercept variance	7.86 (0.23)
Random slope variance	0.01 (0.001)
Intercept-slope covariance	− 0.05 (0.01)
Memory (N = 5,758)	
Time	− **0.11 (0.01), < .001**
Hope	0.01 (0.01), 0.19
Hope X Time	− 0.0004 (0.001), 0.48
Ethnicity	− **1.39 (0.09), < .001**
Sex	**0.41 (0.05), < .001**
Age	− **0.58 (0.02), < .001**
Marital status	
Married/Cohabiting	*REF*
Single	− **0.18 (0.07), 0.01**
Divorced	− 0.11 (0.09), 0.24
Widowed	− 0.12 (0.12), 0.32
Education	**0.04 (0.01), < .001**
Depression	− 0.08 (0.06), 0.20
Random intercept variance	2.42 (0.09)
Random slope variance	0.005 (0.001)
Intercept-slope covariance	− 0.04 (0.007)
Inductive reasoning (N = 5,765)	
Time	− **0.24 (0.03), < .001**
Hope	**0.08 (0.03), 0.004**
Hope X Time	**0.003 (0.001), 0.03**
Ethnicity	− **14.94 (0.46), < .001**
Sex	− **4.33 (0.28), < .001**
Age	− **2.31 (0.10), < .001**
Marital status	
Married/Cohabiting	*REF*
Single	− **1.30 (0.37), < .001**
Divorced	− **0.99 (0.46), 0.03**
Widowed	− 0.94 (0.61), 0.12
Education	**0.43 (0.03), < .001**
Depression	− 0.08 (0.32), 0.80
Random intercept variance	73.92 (1.59)
Random slope variance	0.08 (0.005)
Intercept-slope covariance	− 0.20 (0.07)

^a^ Presented as: b (SE), *p* <.05

The unadjusted models for life satisfaction (Table 4) showed that higher life satisfaction was associated with all baseline cognitive domains (Overall cognition: n = 6,422, b = 0.05, SE = 0.01, *P* < 0.001. Phonemic fluency: n = 6,411, b = 0.20, SE = 0.03, *P* < 0.001. Semantic fluency: n = 6,414, b = 0.17, SE = 0.03, *P* < 0.001. Memory: n = 6,409, b = 0.05, SE = 0.02, *P* = 0.01. Inductive reasoning: n = 6,419, b = 0.87, SE = 0.09, *P* < 0.001). However, life satisfaction was not associated with decline over time in any domain (Overall cognition: b = − 0.0003, SE = 0.0003, *P* = 0.35. Phonemic fluency: b = − 0.002, SE = 0.003, *P* = 0.55. Semantic fluency: b = 0.001, SE = 0.002, *P* = 0.52. Memory: b = − 0.002, SE = 0.002, *P* = 0.13. Inductive reasoning: b = − 0.002, SE = 0.004, *P* = 0.69).

**Table 4:** Unadjusted linear mixed models testing associations between life satisfaction with cognitive function and decline

Overall cognition (N = 6,422)	
Time	− **0.01 (0.002), < .001**^a^
Life satisfaction	**0.05 (0.01), < .001**
Life satisfaction X Time	− 0.0003 (0.0003), 0.35
Random intercept variance	0.51 (0.01)
Random slope variance	0.001 (0.00004)
Intercept-slope covariance	− 0.0001 (0.0005)
Verbal fluency: Phonemic (N = 6,411)	
Time	− **0.09 (0.015), < .001**
Life satisfaction	**0.20 (0.03), < .001**
Life satisfaction X Time	− 0.002 (0.003), 0.55
Random intercept variance	11.53 (0.30)
Random slope variance	0.02 (0.002)
Intercept-slope covariance	− 0.01 (0.02)
Verbal fluency: Semantic (N = 6,414)	
Time	− **0.09 (0.01), < .001**
Life satisfaction	**0.17 (0.03), < .001**
Life satisfaction X Time	0.001 (0.002), 0.52
Random intercept variance	10.31 (0.26)
Random slope variance	0.006 (0.001)
Intercept-slope covariance	− 0.01 (0.01)
Memory (N = 6,409)	
Time	− **0.10 (0.01), < .001**
Life satisfaction	**0.05 (0.02), 0.01**
Life satisfaction X Time	− 0.002 (0.002), 0.13
Random intercept variance	3.00 (0.09)
Random slope variance	0.004 (0.001)
Intercept-slope covariance	− 0.03 (0.007)
Inductive reasoning (N = 6,419)	
Time	− **0.17 (0.02), < .001**
Life satisfaction	**0.87 (0.09), < .001**
Life satisfaction X Time	− 0.002 (0.004), 0.69
Random intercept variance	113.40 (2.20)
Random slope variance	0.08 (0.005)
Intercept-slope covariance	0.12 (0.08)

^a^Presented as: b (SE), *p* <.05

^*^Note: The overall cognition measure is expressed in standard deviation units (due to calculation of z scores), whereas the individual cognitive outcomes are reported using original units

The fully adjusted models for life satisfaction (Table 5) was consistent with the unadjusted model. Specifically, this showed that this was associated with all cognitive domains at analytic baseline (Overall cognition: n = 5,772, b = 0.04, SE = 0.01, *P* < 0.001. Phonemic fluency: n = 5,765, b = 0.19, SE = 0.03, *P* < 0.001. Semantic fluency: n = 5,768, b = 0.14, SE = 0.03, *P* < 0.001. Memory: n = 5,764, b = 0.04, SE = 0.02, *P* = 0.02. Inductive reasoning: n = 5,771, b = 0.60, SE = 0.08, *P* < 0.001). However, there were no associations between life satisfaction and rate of decline in any cognitive domain (Overall cognition: b = − 0.0001, SE = 0.0004, *P* = 0.67. Phonemic fluency: b = − 0.002, SE = 0.003, *P* = 0.46. Semantic fluency: b = 0.002, SE = 0.002, *P* = 0.46. Memory: b = − 0.001, SE = 0.002, *P* = 0.63. Inductive reasoning: b = 0.001, SE = 0.004, *P* = 0.81).

**Table 5:** Adjusted linear mixed models testing associations between life satisfaction with cognitive function and decline

Overall Cognition (n = 5,772)	
Time	− **0.01 (0.002), < .001**^**a**^
Life satisfaction	**0.04 (0.01), < .001**
Life satisfaction X Time	− 0.0001 (0.0004), 0.67
Ethnicity	− **0.89 (0.03), < .001**
Sex	− 0.04 (0.02), 0.06
Age	− **0.22 (0.01), < .001**
Marital status	
Married/Cohabiting	*REF*
Single	− **0.11 (0.03), < .001**
Divorced	− **0.07 (0.03), 0.02**
Widowed	− **0.10 (0.04), 0.02**
Education	**0.03 (0.002), < .001**
Depression	0.01 (0.02), 0.60
Random intercept variance	0.36 (0.008)
Random slope variance	0.0005 (0.00003)
Intercept-slope covariance	− 0.003 (0.0004)
Verbal fluency: Phonemic (N = 5,765)	
Time	− **0.08 (0.02), < .001**
Life satisfaction	**0.19 (0.03), < .001**
Life satisfaction X Time	− 0.002 (0.003), 0.46
Ethnicity	− **2.78 (0.18), < .001**
Sex	**0.24 (0.11), 0.02**
Age	− **0.84 (0.04), < .001**
Marital status	
Married/Cohabiting	*REF*
Single	− **0.70 (0.14), < .001**
Divorced	− **0.38 (0.18), 0.03**
Widowed	− **0.61 (0.23), 0.01**
Education	**0.10 (0.01), < .001**
Depression	0.11 (0.12), 0.36
Random intercept variance	9.70 (0.28)
Random slope variance	0.02 (0.002)
Intercept-slope covariance	− 0.06 (0.02)
Verbal fluency: Semantic (N = 5,768)	
Time	− **0.09 (0.01), < .001**
Life satisfaction	**0.14 (0.03), < .001**
Life satisfaction X Time	0.002 (0.002), 0.46
Ethnicity	− **3.86 (0.16), < .001**
Sex	0.09 (0.09), 0.37
Age	− **0.88 (0.03), < .001**
Marital status	
Married/Cohabiting	*REF*
Single	− **0.49 (0.13), < .001**
Divorced	− **0.37 (0.16), 0.02**
Widowed	− **0.49 (0.21), 0.02**
Education	**0.11 (0.01), < .001**
Depression	0.10 (0.11), 0.34
Random intercept variance	7.75 (0.23)
Random slope variance	0.01 (0.001)
Intercept-slope covariance	− 0.04 (0.01)
Memory (N = 5,764)	
Time	− **0.11 (0.01), < .001**
Life satisfaction	**0.04 (0.02), 0.02**
Life satisfaction X Time	− 0.001 (0.002), 0.63
Ethnicity	− **1.39 (0.09), < .001**
Sex	**0.41 (0.05), < .001**
Age	− **0.58 (0.02), < .001**
Marital status	
Married/Cohabiting	*REF*
Single	− **0.17 (0.07), 0.02**
Divorced	− 0.09 (0.09), 0.31
Widowed	− 0.11 (0.12), 0.33
Education	**0.04 (0.01), < .001**
Depression	− 0.06 (0.06), 0.33
Random intercept variance	2.42 (0.09)
Random slope variance	0.005 (0.001)
Intercept-slope covariance	− 0.04 (0.007)
Inductive reasoning (N = 5,771)	
Time	− **0.18 (0.03), < .001**
Life satisfaction	**0.60 (0.08), < .001**
Life satisfaction X Time	0.001 (0.004), 0.81
Ethnicity	− **14.83 (0.46), < .001**
Sex	− **4.44 (0.28), < .001**
Age	− **2.35 (0.10), < .001**
Marital status	
Married/Cohabiting	*REF*
Single	− **1.05 (0.37), 0.004**
Divorced	− 0.59 (0.47), 0.21
Widowed	− 0.68 (0.60), 0.26
Education	**0.43 (0.03), < .001**
Depression	0.26 (0.31), 0.40
Random intercept variance	73.79 (1.59)
Random slope variance	0.07 (0.005)
Intercept-slope covariance	− 0.21 (0.07)

^a^ Presented as: b (SE), *p* <.05

### Supplementary analyses

Supplementary analyses were conducted excluding people with a diagnosis of dementia at any point during follow up and excluding people who died during follow up, results were largely similar to main results. Outputs are presented in Supplementary Materials (Supplementary Tables 2, 3). All main associations remained consistent when using the categorical age bands and their interaction with time in models (Supplementary Table 4).

#### Sex interactions

Analyses including sex interaction terms showed that there were no sex differences in the association between hope and cognitive function (b = − 0.004, SE = 0.004, P = 0.35). However, there was a significant sex interaction for the life satisfaction model (b = − 0.02, SE = 0.01, P = 0.03). Stratified models showed that higher life satisfaction was associated with better cognitive function at baseline in both men and women, though this association is stronger in men. Both models showed no association between life satisfaction and cognitive decline over time (Supplementary Table 5).

#### Age interactions

There was a significant age interaction in the association between hope and cognitive function by age (b = 0.004, SE = 0.001, P = 0.007). Stratified models showed that there was no significant association between hope and cognitive function or decline in younger people (age < 65). However, hope was significantly associated with cognitive function in older people (age 65 +), but not cognitive decline (Supplementary Table 6).

Similarly, there was a significant age interaction for life satisfaction (b = 0.01, SE = 0.004, P = 0.005). Stratified models showed that there was a significant association between life satisfaction and cognitive function for both younger and older people, but the association was stronger in the older group. There were no significant associations between life satisfaction and cognitive decline in either group (Supplementary Table 6).

## Discussion

The aim of this study was to test associations between life satisfaction and hope with cognitive function and decline across middle to older adulthood. This study suggests that life satisfaction is associated with higher cognitive function (overall cognition, verbal fluency, memory and inductive reasoning) at analytic baseline, but not the rate of decline over time. These findings suggest that in this sample, people with lower life satisfaction show lower initial cognitive function and this difference is maintained over time. This is important because although decline is no steeper for those with lower life satisfaction, they may reach the clinical threshold for dementia earlier than those with higher life satisfaction. This association existed for both men and women, but was stronger in men. Similarly, associations were observed across age groups, but associations were significantly stronger in older people (age 65 +) compared with younger (< 65).

Hope was also associated with cognitive function (overall cognition, phonemic verbal fluency, and inductive reasoning) at analytic baseline. There were significant age differences in the association between hope and overall cognitive function, whereby the association was observed in the older group (age 65 +) but not in the younger group (< 65). Hope was not associated with semantic verbal fluency or memory at analytic baseline. Higher levels of hope were associated with slower decline in inductive reasoning (but not other cognitive functions) over the 13-year follow up, even after adjustment for depression and other key covariates. Inductive reasoning is related to many instrumental activities of daily living associated with maintaining independence in older age (Boron 2005). Research has also shown inductive reasoning to be an important predictor of individual differences in everyday cognitive functioning over time in older people (Yam et al. 2014). The finding that hope may play a role in maintaining inductive reasoning over time is therefore important.

This study showed that effects of hope on cognitive function over time were limited to inductive reasoning (though the effect was small), and no associations were observed with rate of decline in any other cognitive domain. The precise underlying mechanism of this association cannot be determined from this study, and should be the subject of future research. It should be noted that the coefficients observed in this study were small, which may limit public health impact. However, small associations may still have an impact at the population level and be informative in the development of multi-domain predictive models for cognitive ageing and dementia, in combination with other risk and protective factors.

### Comparison to previous literature

These findings are consistent with previous research showing cross-sectional associations between life satisfaction and cognitive function (Jones et al. 2003; Requena et al. 2009; Zahodne et al. 2018), and with longitudinal research showing life satisfaction is not associated with rate of decline in cognition over time (Nakanishi et al. 2019; Ihle et al. 2021). The findings are not consistent with research showing associations between life satisfaction and slower decline in perceptual speed (Gerstorf et al. 2007). This may be because of differences in sample and study methodologies, and in particular the life satisfaction assessment used between studies. In this study, life satisfaction was measured using a single item asking participants about their satisfaction with life as a whole. By contrast, Gerstorf et al. (2007) used a scale comprising three dimensions, including life satisfaction (4 items), satisfaction with ageing (5 items) and non-agitation (6 items). It is therefore plausible that longitudinal associations with cognition in the Gerstorf et al. (2007) study may have been primarily driven by the satisfaction with ageing or non-agitation dimension of the measure. In addition, the two studies also differed in cognitive domain assessed, with this study focusing on verbal fluency, memory, and inductive reasoning, and the Gerstorf et al. (2007) focusing on perceptual speed. It is therefore plausible that differential associations may exist between life satisfaction and different cognitive functions.

Previous research has shown that hopelessness is associated with risk of cognitive impairment (Håkansson et al. 2015). However, there has been limited research investigating hope or hopelessness in relation to cognitive decline prior to onset of impairment. This study extends previous findings by showing that even after adjusting for depression, hope is associated with baseline cognitive function (particularly phonemic fluency and inductive reasoning). The study also extends previous research by showing hope is associated with slower decline in inductive reasoning over time.

### Limitations

Several limitations should be acknowledged when interpreting results. First, a single item measure of overall life satisfaction was used, meaning this study could not test for differential effects of satisfaction in specific areas of life (e.g. relationships, work, health, etc.) on cognitive outcomes. The use of a single measure of satisfaction with life as a whole, rather than a more comprehensive assessment, may have masked individual effects of particular domains of life satisfaction. The study also relies on single measures of life satisfaction and hope available at analytic baseline, assuming that these constructs are stable over time. However, these traits may fluctuate and vary over the life course, for example in response to life events and health factors. The use of single time-point measures for life satisfaction and hope in this study may therefore not fully capture the dynamic nature of these traits over time and how this is associated with cognitive function across the life course. If baseline measures were not stable, this may have introduced measurement error, in turn potentially attenuating associations. As such, associations observed in this study may be conservative estimates. Future research should aim to address this by incorporating repeated measures of wellbeing over time.

In addition to this, though Whitehall II comprises a reasonably large sample, it is not representative of the general population. Specifically, the sample includes people who were employed by the British Civil Service in 1985–1988, meaning that particular demographic groups are under-represented in this sample. For example, only 27.76% of the sample were women and only 7.02% were from minority ethnic backgrounds. A previous study has shown that a range of key factors are associated with non-response and withdrawal over time in the Whitehall II study (Mein et al. 2012). The current study used data from wave 7 onwards, meaning that generalisability of the sample is likely affected by this attrition and selection bias.

Additionally, although people with dementia were excluded from supplementary analyses to ensure this group did not exert a disproportionate effect on results, we were not able to exclude people with undiagnosed dementia or cognitive impairment short of dementia. In addition, ethnicity was coded as white or non-white, meaning that minority racial and ethnic groups were combined into a single category. This is a limitation because it collapses across heterogeneous racial and ethnic identities with distinct lived experiences and differing rates of cognitive decline and dementia.

To maintain model parsimony, interactions between covariates and time were not included in analyses, meaning time-varying effects of covariates were not considered. It is also possible that age or sex may differentially influence these associations. While interactions and stratified models were run for overall cognitive function, future research could consider potential interactions across individual cognitive domains. Next causality cannot be inferred from these results, as causal inference methodologies were not used. Future research should use causal inference methodologies to test whether there is a causal link between different aspects of wellbeing and cognitive decline. Related to this, future research could also test whether intervention to improve wellbeing in older people is associated with cognitive benefits.

## Conclusion

In conclusion, findings from this study show that life satisfaction was associated with baseline cognitive function, but not rate of decline over a period of 13 years. Hope was associated with baseline phonemic fluency and inductive reasoning and with slower decline in inductive reasoning over 13 years. These findings improve current understanding of the temporal relationship between cognitive function and aspects of wellbeing for which existing evidence is mixed or limited.

## Supplementary Information

Below is the link to the electronic supplementary material.

Supplementary file1 (DOCX 34 KB)

## Data Availability

Data are available upon application to the Whitehall II team.
