# Supplementary material for: Associations between life satisfaction and hope with cognitive function and decline over 13 years: findings from the Whitehall II study
Source: Eur J Ageing. 2025 Nov 25;22(1):59. doi: 10.1007/s10433-025-00892-8 (PMC12647409; doi:10.1007/s10433-025-00892-8)
Supplement: Supplementary file 1 — Supplementary file1 (DOCX 34 KB) [file 10433_2025_892_MOESM1_ESM.docx]

**SUPPLEMENTARY MATERIALS**

**Article Title:** Associations between life satisfaction and hope with cognitive function and decline over 13 years: Findings from the Whitehall II study.

**Journal Name:** European Journal of Ageing.

**Author names:** Amber John^a^, Aysha Mohamed Rafik Patel^a^, Roopal Desai^a^, Emily Willroth^b^, Natalie L. Marchant^c^, Harriet Demnitz-King^c^, Barbara Woodward-Carlton, Dorina Cadar^d^, David Bartres-Faz^e^, Rob Saunders^f^, Georgia Bell^a^, Aida Suarez Gonzalez^g^, Darya Gaysina^h^, Marcus Richards^i^, Joshua Stott^a^.

**Corresponding Author:** Dr Amber John, ADAPT Lab, Research Department of Clinical, Educational and Health Psychology, UCL, London, UK; Tel: 02031085868; Email: A.john@ucl.ac.uk; Twitter: @ADAPTLabUCL.

**Supplementary Table 1:** Comparing key variables and covariates between the analytic sample and the sample excluded due to missing data.

**Supplementary Table 2:** Linear mixed models testing associations between hope and life satisfaction with cognitive function and decline, excluding people with dementia

**Supplementary Table 3:** Linear mixed models testing associations between hope and life satisfaction with cognitive function and decline, excluding people who died during follow up

**Supplementary Table 4:** Linear mixed models testing associations between hope and life satisfaction with cognitive function and decline, including age as categorical age bands and their interactions with time.

**Supplementary Table 5:** Models stratified by sex.

**Supplementary Table 6:** Models stratified by age group.

**Supplementary Table 1: Comparing key variables and covariates between the analytic sample and the sample excluded due to missing data.**

|  | **Sample included in fully adjusted analytic models N=5,716** | **Sample excluded due to missing data  N=4,595** | **Difference (X2 or t-test)** |
| --- | --- | --- | --- |
| Age, N (%) |  |  |  |
| 50-54 | 1,063 (18.60) | 180 (14.72) | **33.61 (4), <.001** |
| 55-59 | 1,728 (30.23) | 320 (26.17) |  |
| 60-64 | 1,220 (21.34) | 270 (22.08) |  |
| 65-69 | 1,164 (10.36) | 293 (23.96) |  |
| 70-74 | 541 (9.46) | 160 (13.08) |  |
| Sex, N (%) |  |  |  |
| Male | 4,129 (72.24) | 2,766 (60.24) | **165.58 (1), <.001** |
| Female | 1,587 (27.76) | 1,826 (39.76) |  |
| Ethnicity, N (%) |  |  |  |
| White | 5,315 (92.98) | 3,866 (85.91) | **138.36 (1), <.001** |
| Ethnic minority | 401 (7.02) | 634 (14.09) |  |
| Marital status, N (%) |  |  |  |
| Married/Cohabiting | 4,363 (76.33) | 853 (71.02) | **18.33 (3), <.001** |
| Single | 709 (12.40) | 166 (13.82) |  |
| Divorced | 406 (7.10) | 110 (9.16) |  |
| Widowed | 238 (4.16) | 72 (6.00) |  |
| Education, Mean (SD) | 15.03 (4.17) | 14.48 (4.62) | **-4.26 (7089), <.001** |
| Depression, N (%) |  |  |  |
| No | 4,573 (80.00) | 715 (75.11) | **11.93 (1), .001** |
| Yes | 1,143 (20.00) | 237 (24.89) |  |
| W7 Overall cognition, Mean (SD) | 0.04 (0.75) | -0.29 (0.90) | **-11.22 (63680, <.001** |
| W7 Words, Mean (SD) | 15.83 (4.10) | 14.86 (4.74) | **-6.18 (6330), <.001** |
| W7 Animals, Mean (SD) | 15.71 (3.83) | 14.50 (4.23) | **-8.25 (6345), <.001** |
| W7 Memory, Mean (SD) | 6.82 (2.36) | 6.35 (2.47) | **-5.19 (6327), <.001** |
| W7 Ah4, Mean (SD) | 44.34 (10.79) | 38.17 (13.32) | **-14.70 (6360), <.001** |
| Hope, Mean (SD) | 18.57 (4.57) | 17.87 (4.98) | **-4.39 (6690), <.001** |
| Life satisfaction, Mean (SD) | 5.58 (1.57) | 5.32 (1.71) | **-4.75 (6714), <.001** |

**Supplementary Table 2:** Linear mixed models testing associations between hope and life satisfaction with cognitive function and decline, excluding people with dementia

|  | **Overall Cognition** | **Verbal fluency: Phonemic** | **Verbal fluency: Semantic** | **Memory** | **Inductive reasoning** |
| --- | --- | --- | --- | --- | --- |
| **Hope** |  |  |  |  |  |
| **N** | **5,663** | **5,659** | **5,659** | **5,656** | **5,662** |
| Time | **-0.01 (0.002), <.001** | **-0.09 (0.02), <.001** | **-0.09 (0.01), <.001** | **-0.11 (0.01), <.001** | **-0.23 (0.03), <.001** |
| Hope | **0.01 (0.002), 0.01** | **0.04 (0.01), 0.004** | 0.01 (0.01), 0.52 | 0.01 (0.01), 0.26 | **0.08 (0.03), 0.01** |
| Hope X Time | 0.0001 (0.0001), 0.68 | -0.0002 (0.001), 0.79 | 0.001 (0.001), 0.49 | -0.0004 (0.001), 0.52 | **0.003 (0.001), 0.05** |
| Ethnicity | **-0.91 (0.03), <.001** | **-2.80 (0.18), <.001** | **-3.91 (0.16), <.001** | **-1.43 (0.09), <.001** | **-15.11 (0.47), <.001** |
| Sex | -0.03 (0.02), 0.12 | **0.27 (0.11), 0.01** | 0.11 (0.09), 0.25 | **0.41 (0.05), <.001** | **-4.30 (0.28), <.001** |
| Age | **-0.21 (0.01), <.001** | **-0.80 (0.04), <.001** | **-0.84 (0.03), <.001** | **-0.56 (0.02), <.001** | **-2.21 (0.10), <.001** |
| Marital status |  |  |  |  |  |
| Married/Cohabiting | *REF* | *REF* | *REF* | *REF* | *REF* |
| Single | **-0.13 (0.03), <.001** | **-0.78 (0.14), <.001** | **-0.58 (0.13), <.001** | **-0.17 (0.07), 0.01** | **-1.35 (0.37), <.001** |
| Divorced | **-0.10 (0.03), 0.002** | **-0.46 (0.18), 0.01** | **-0.48 (0.16), 0.003** | -0.11 (0.09), 0.23 | **-1.11 (0.46), 0.02** |
| Widowed | **-0.09 (0.04), 0.04** | **-0.58 (0.24), 0.02** | **-0.46 (0.21), 0.03** | -0.10 (0.12), 0.42 | -0.83 (0.62), 0.18 |
| Education | **0.03 (0.002), <.001** | **0.11 (0.01), <.001** | **0.11 (0.01), <.001** | **0.04 (0.01), <.001** | **0.43 (0.03), <.001** |
| Depression | -0.01 (0.02), 0.73 | 0.05 (0.13), 0.70 | -0.03 (0.11), 0.80 | -0.07 (0.06), 0.25 | -0.07 (0.33), 0.84 |
| **Life satisfaction** |  |  |  |  |  |
| **N** | **5,667** | **5,662** | **5,664** | **5,660** | **5,666** |
| Time | **-0.01 (0.002), <.001** | **-0.08 (0.02), <.001** | **-0.09 (0.01), <.001** | **-0.11 (0.01), <.001** | **-0.18 (0.02), <.001** |
| Life satisfaction | **0.04 (0.01), <.001** | **0.18 (0.03), <.001** | **0.13 (0.03), <.001** | **0.04 (0.02), 0.03** | **0.58 (0.08), <.001** |
| Life satisfaction X Time | -0.0001 (0.0004), 0.79 | -0.002 (0.003), 0.51 | 0.002 (0.002), 0.39 | -0.001 (0.002), 0.73 | 0.001 (0.004), 0.73 |
| Ethnicity | **-0.91 (0.03), <.001** | **-2.80 (0.18), <.001** | **-3.88 (0.16), <.001** | **-1.42 (0.09), <.001** | **-15.00 (0.47), <.001** |
| Sex | -0.03 (0.02), 0.08 | **0.26 (0.11), 0.02** | 0.10 (0.09), 0.31 | **0.41 (0.05), <.001** | **-4.39 (0.28), <.001** |
| Age | **-0.22 (0.01), <.001** | **-0.82 (0.04), <.001** | **-0.85 (0.03), <.001** | **-0.56 (0.02), <.001** | **-2.26 (0.10), <.001** |
| Marital status |  |  |  |  |  |
| Married/Cohabiting | *REF* | *REF* | *REF* | *REF* | *REF* |
| Single | **-0.12 (0.03), <.001** | **-0.72 (0.14), <.001** | **-0.53 (0.13), <.001** | **-0.16 (0.07), 0.02** | **-1.10 (0.37), 0.003** |
| Divorced | **-0.08 (0.03), 0.02** | **-0.41 (0.18), 0.03** | **-0.39 (0.16), 0.02** | -0.09 (0.09), 0.30 | -0.71 (0.47), 0.13 |
| Widowed | -0.08 (0.04), 0.06 | **-0.58 (0.24), 0.01** | -0.41 (0.21), 0.05 | -0.09 (0.12), 0.44 | -0.58 (0.61), 0.35 |
| Education | **0.03 (0.002), <.001** | **0.11 (0.01), <.001** | **0.11 (0.01), <.001** | **0.04 (0.01), <.001** | **0.44 (0.03), <.001** |
| Depression | 0.01 (0.02), 0.49 | 0.12 (0.12), 0.32 | 0.11 (0.11), 0.31 | -0.05 (0.06), 0.43 | 0.31 (0.31), 0.32 |

**Supplementary Table 3:** Linear mixed models testing associations between hope and life satisfaction with cognitive function and decline, excluding people who died during follow up

|  | **Overall Cognition** | **Verbal fluency: Phonemic** | **Verbal fluency: Semantic** | **Memory** | **Inductive reasoning** |
| --- | --- | --- | --- | --- | --- |
| **Hope** |  |  |  |  |  |
| **N** | **5,199** | **5,198** | **5,197** | **5,196** | **5,199** |
| Time | **-0.01 (0.002), <.001** | **-0.09 (0.02), <.001** | **-0.09 (0.02), <.001** | **-0.11 (0.01), <.001** | **-0.23 (0.03), <.001** |
| Hope | 0.004 (0.002), 0.10 | **0.03 (0.01), 0.01** | 0.002 (0.01), 0.86 | 0.002 (0.01), 0.72 | 0.05 (0.03), 0.09 |
| Hope X Time | 0.0001 (0.0001), 0.60 | -0.0002 (0.001), 0.83 | 0.001 (0.001), 0.49 | -0.0001 (0.001), 0.85 | **0.003 (0.002), 0.03** |
| Ethnicity | **-0.93 (0.03), <.001** | **-2.93 (0.19), <.001** | **-4.04 (0.17), <.001** | **-1.41 (0.09), <.001** | **-15.37 (0.48), <.001** |
| Sex | -0.03 (0.02), 0.11 | **0.26 (0.11), 0.02** | 0.12 (0.10), 0.24 | **0.41 (0.06), <.001** | **-4.30 (0.29), <.001** |
| Age | **-0.21 (0.01), <.001** | **-0.79 (0.04), <.001** | **-0.84 (0.03), <.001** | **-0.56 (0.02), <.001** | **-2.21 (0.10), <.001** |
| Marital status |  |  |  |  |  |
| Married/Cohabiting | *REF* | *REF* | *REF* | *REF* | *REF* |
| Single | **-0.14 (0.03), <.001** | **-0.79 (0.15), <.001** | **-0.55 (0.13), <.001** | **-0.20 (0.07), 0.01** | **-1.56 (0.38), <.001** |
| Divorced | **-0.09 (0.03), 0.01** | **-0.44 (0.19), 0.02** | **-0.43 (0.17), 0.01** | -0.10 (0.09), 0.30 | **-1.02 (0.49), 0.04** |
| Widowed | **-0.12 (0.05), 0.01** | **-0.64 (0.25), 0.01** | **-0.61 (0.22), 0.01** | -0.16 (0.13), 0.20 | -1.03 (0.65), 0.11 |
| Education | **0.03 (0.002), <.001** | **0.11 (0.01), <.001** | **0.11 (0.01), <.001** | **0.04 (0.01), <.001** | **0.42 (0.03), <.001** |
| Depression | -0.01 (0.02), 0.69 | 0.06 (0.13), 0.64 | -0.03 (0.11), 0.78 | -0.10 (0.06), 0.13 | 0.02 (0.34), 0.96 |
| **Life satisfaction** |  |  |  |  |  |
| **N** | **5,199** | **5,198** | **5,198** | **5,196** | **5,199** |
| Time | **-0.01 (0.002), .004** | **-0.08 (0.02), <.001** | **-0.09 (0.01), <.001** | **-0.11 (0.01), <.001** | **-0.19 (0.03), <.001** |
| Life satisfaction | **0.04 (0.01), <.001** | **0.19 (0.04), <.001** | **0.12 (0.03), <.001** | 0.04 (0.02), 0.05 | **0.55 (0.09), <.001** |
| Life satisfaction X Time | -0.0002 (0.0004), 0.61 | -0.002 (0.003), 0.38 | 0.001 (0.002), 0.52 | -0.001 (0.002), 0.69 | 0.002 (0.004), 0.58 |
| Ethnicity | **-0.92 (0.03), <.001** | **-2.87 (0.19), <.001** | **-4.00 (0.17), <.001** | **-1.40 (0.09), <.001** | **-15.16 (0.48), <.001** |
| Sex | -0.03 (0.02), 0.09 | **0.26 (0.11), 0.02** | 0.11 (0.10), 0.27 | **0.41 (0.06), <.001** | **-4.37 (0.29), <.001** |
| Age | **-0.22 (0.01), <.001** | **-0.80 (0.04), <.001** | **-0.85 (0.03), <.001** | **-0.56 (0.02), <.001** | **-2.25 (0.10), <.001** |
| Marital status |  |  |  |  |  |
| Married/Cohabiting | *REF* | *REF* | *REF* | *REF* |  |
| Single | **-0.13 (0.03), <.001** | **-0.75 (0.15), <.001** | **-0.50 (0.13), <.001** | **-0.19 (0.07), 0.01** | **-1.31 (0.38), 0.001** |
| Divorced | **-0.07 (0.03), 0.04** | -0.37 (0.19), 0.05 | **-0.35 (0.17), 0.04** | -0.08 (0.09), 0.40 | -0.64 (0.49), 0.19 |
| Widowed | **-0.11 (0.05), 0.02** | **-0.64 (0.25), 0.01** | **-0.57 (0.22), 0.01** | -0.15 (0.13), 0.25 | -0.73 (0.65), 0.26 |
| Education | **0.03 (0.002), <.001** | **0.11 (0.01), <.001** | **0.11 (0.01), <.001** | **0.04 (0.01), <.001** | **0.42 (0.03), <.001** |
| Depression | 0.02 (0.02), 0.45 | 0.14 (0.12), 0.25 | 0.11 (0.11), 0.32 | -0.06 (0.06), 0.34 | 0.41 (0.32), 0.20 |

**Supplementary Table 4:** Linear mixed models testing associations between hope and life satisfaction with cognitive function and decline, including age as categorical age bands and their interactions with time.

| **Hope** | **N=5,766** | **Life satisfaction** | **N=5,772** |
| --- | --- | --- | --- |
| Time | -0.002 (0.002), .48 | Time | -0.001 (0.002), .59 |
| Hope | **0.005 (0.002), 0.01** | Life satisfaction | **0.03 (0.01), <.001** |
| Hope X Time | 0.0002 (0.0001), 0.16 | Life satisfaction X Time | 0.0005 (0.0003), 0.18 |
| Age |  | Age |  |
| 50-54 | *REF* | 50-54 | *REF* |
| 55-59 | **-0.17 (0.03), <.001** | 55-59 | -0.17 (0.03), <.001 |
| 60-64 | **-0.32 (0.03), <.001** | 60-64 | -0.33 (0.03), <.001 |
| 65-69 | **-0.52 (0.03), <.001** | 65-69 | -0.53 (0.03), <.001 |
| 70-74 | **-0.68 (0.04), <.001** | 70-74 | -0.69 (0.04), <.001 |
| Age X Time |  | Age X Time |  |
| 50-54 | *REF* | 50-54 | *REF* |
| 55-59 | **-0.004 (0.002), .02** | 55-59 | **-0.004 (0.002), .02** |
| 60-64 | **-0.01 (0.002), <.001** | 60-64 | **-0.01 (0.002), <.001** |
| 65-69 | **-0.03 (0.002), <.001** | 65-69 | **-0.03 (0.002), <.001** |
| 70-74 | **-0.03 (0.002), <.001** | 70-74 | **-0.03 (0.002), <.001** |
| Ethnicity | **-0.90 (0.03), <.001** | Ethnicity | **-0.90 (0.03), <.001** |
| Sex | -0.03 (0.02), 0.09 | Sex | -0.04 (0.02), 0.05 |
| Marital status |  | Marital status |  |
| Married/Cohabiting | *REF* | Married/Cohabiting | *REF* |
| Single | **-0.13 (0.03), <.001** | Single | **-0.11 (0.03), <.001** |
| Divorced | **-0.09 (0.03), 0.004** | Divorced | **-0.07 (0.03), 0.02** |
| Widowed | **-0.11 (0.04), 0.01** | Widowed | **-0.10 (0.04), 0.02** |
| Education | **0.03 (0.002), <.001** | Education | **0.03 (0.002), <.001** |
| Depression | -0.01 (0.02), 0.65 | Depression | 0.01 (0.02), 0.48 |

**Supplementary Table 5:** Models stratified by sex.

| **LIFE SATISFACTION** |  |
| --- | --- |
| **Men** | |
| Life satisfaction | 0.04, (0.007), <.001 |
| Life satisfaction X Time | -0.000, (0.000), .71 |
| **Women** | |
| Life satisfaction | 0.02, (0.01), .03 |
| Life satisfaction X Time | -0.000, (0.001), .97 |

*Stratified models for hope not run, as there was no significant sex interaction.

**Supplementary Table 6:** Models stratified by age group.

| **HOPE** |  |
| --- | --- |
| **Younger <65** | |
| Hope | 0.002, (0.002), .44 |
| Hope X Time | 0.000, (0.000), .09 |
| **Older >65** | |
| Hope | 0.01, (0.004), .03 |
| Hope X Time | -0.000, (0.000), .25 |
| **LIFE SATISFACTION** |  |
| **Younger <65** | |
| Life satisfaction | 0.02, (0.007), .003 |
| Life satisfaction X Time | 0.000, (0.000), .40 |
| **Older >65** | |
| Life satisfaction | 0.05, (0.01), .001 |
| Life satisfaction X Time | -0.000, (0.001), .82 |
